# Significantly hypoglycemic effect of a novel functional bread rich in mulberry bark and improving the related functions of liver, pancreas, and kidney, on T2D mice

**DOI:** 10.1002/fsn3.2189

**Published:** 2021-03-08

**Authors:** Yu‐Jie Weng, Meng Zhang, Jiang Wang, Yu‐Qing Zhang

**Affiliations:** ^1^ School of Biology and Basic Medical Sciences Soochow University Suzhou China; ^2^ National Engineering Laboratory for Modern Silk Soochow University Suzhou China

**Keywords:** bread, hypoglycemic effect, mulberry branch bark powder, T2D mice

## Abstract

To develop a novel functional food with lowering blood glucose for diabetics, the mixed bread containing mulberry branch bark powder (MBBP) was used for the oral administration of the type 2 diabetic (T2D) mice induced by streptozocin (STZ), high‐fat and high‐sugar diet for 7 weeks. 5%, 10%, and 15% MBBP bread diets had a significatively positive influence on the biochemical indicators, histological examination, and immunohistochemistry observations in T2D mice. The 15% MBBP‐rich bread diet evidently retarded loss weight of T2D mice, and decreased in FBG by about 55% and in glycosylated hemoglobin (HbA1c) levels by about 30%. Its glucose tolerance and serum insulin levels were very close to normal level. The abnormal lipid metabolism and insulin‐related index of T2D mice in both the 10% and 15% MBBP bread diet groups were partly reversed. The Western blotting results showed that the expression levels of key proteins in PI3K/AKT signaling pathway were decreased and expression levels of immunoproteins PPARγ, TNF‐α, P65, and IL6 were increased. In general, oral MBBP bread diets effectively restored some functions and repaired damage to the pancreas, liver, and kidney in T2D mice. Therefore, MBBP is potential to develop into a novel functional food additive with significantly hypoglycemic effect.

## Graphical abstract

The mulberry branches (left) were peeled and its barks were pulverized into ultrafine bark powder (middle). A novel bread (right) containing 5% ~ 15% bark powder has a significantly hypoglycemic effect and can improve the related functions of liver, pancreas, and kidney in T2D mice.

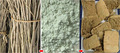

## 1 INTRODUCTION

Diabetes mellitus is a high‐incidence chronic disease, which is characterized by high blood glucose levels, low body weights, and insulin secretion increase. From 2010 to 2030, number with diabetes around the world is predicted to increase 54%, and thirty‐six percent is predicted to take place in China and India (Shaw et al., 2010). Therefore, the treatment of diabetes is raising more and more attention. The current treatment ways are generally taking medicine combined with controlling diet. As far as DM drugs are concerned, the medicine which mainly consists of chemical compounds have serious side effects in long‐term using. Therefore, traditional Chinese medicines are much accounted of stable efficacy, low toxicity, fewer adverse treatment ways reactions (Liang et al., 2015; Ling, 2015). As far diet, people with diabetes are required to reduce intake of carbohydrates in daily life. Hence, people with diabetes often feel hungry. In view of the above two situations, we want to develop a new type of food that can show better both in large quantities and in the treatment of diabetes.

Mulberry (*Morus* L.) has been a traditional Chinese medicine since ancient China. The annual yield of mulberry branches is quite large, but mulberry branches utilization rate is low. In recent years, plenty of studies have shown that mulberry branches contain many bioactive components including alkaloids, phenols, polysaccharides, and flavonoids (Qiu et al., 2016), mulberry branches also have rich medicinal value including hypoglycemic (Wu et al., 2005; Ye et al., 2002; Wang et al., 2014), hypolipidemic (He et al., 2007), and antioxidant activities (Mazimba et al., 2011) et al. The development of medicinal value of mulberry is the key to mulberry utilization.

Diabetics cannot consume too much carbohydrates. In clinical treatment, diabetics usually are allowed to keep a low carbohydrate diet which means carbohydrate intake should between 30–200 g/day (Wang et al., 2018). This causes diabetes to feel hungry at times. Man has a long history of making and eating bread all over the world (Samuel, 1996). Bread is made from wheat flour and contains a lot of carbohydrates. Therefore, diabetes is not ought to eat a lot of bread. Now, researchers have been studying bread that has a hypoglycemic effect (Waghmare and Arya, 2014).

Our laboratory has studied the effects of mulberry branch bark powder (MBBP) both as Chinese medicine and as food supplement on diabetes (Liu et al., 2016). The ethanol extract of MBBP could regulate the mRNA expression of glycometabolism genes to regulate sugar metabolism and reduce the blood glucose level in T2D mice. It could also increase the mRNA expression of the genes those could help decrease the insulin resistance in T2D mice (Liu et al., 2014). Through the results of metabolic pathways by GC‐MS in T2D mice, oral administration of MBBP could ameliorate the hyperglycemia and hyperlipidemia symptoms on unitary level and have a dosage‐dependent effect on restoring the abnormal metabolic state to a near normal level (Qiu and Zhang, 2019). In this experiment, MBBP was added into bread to make MBBP‐bread, and then fed to T2D mice induced by combined STZ with high fat and high glucose diet. Finally, therapeutic effects of MBBP‐bread on mice with type 2 diabetes were going to be explored.

## 2 MATERIALS AND METHODS

### 2.1 MBBP and MBBP‐bread preparation

The *Ramulus mori* branches of the mulberry (*HuSang 32, M. multicaulis* L.) were provided by the Mulberry Garden of Soochow University, Suzhou, China, in November 2018. These barks which were peeled from mulberry branches, then were placed in the oven and dried at 100°C for 2 hr. Pulverized into powder twice to make sure it could pass through a 100‐mesh sieve. According to Table 1, bread mechan (Philips, HD9016) was used to make normal bread and MBBP‐bread.

**TABLE 1:** Basic formula of normal bread and MBBP‐breads (bread quality: 1,000 g)

Constituent	Quality (g) or Volume (ml)			
	Normal bread	5% MBBP‐bread	10% MBBP‐bread	15% MBBP‐bread
Bread powder	560.00	510.00	460.00	410.00
MBBP	0.00	50.00	100.00	150.00
Water	400.00	400.00	400.00	400.00
Oil	30.00	30.00	30.00	30.00
Salt	7.50	7.50	7.50	7.50
Yeast	3.00	3.00	3.00	3.00

Note

The amount of MBBP added is based on the total quality of bread.

### 2.2 MBBP‐bread fodder preparation

**FIGURE 1:**
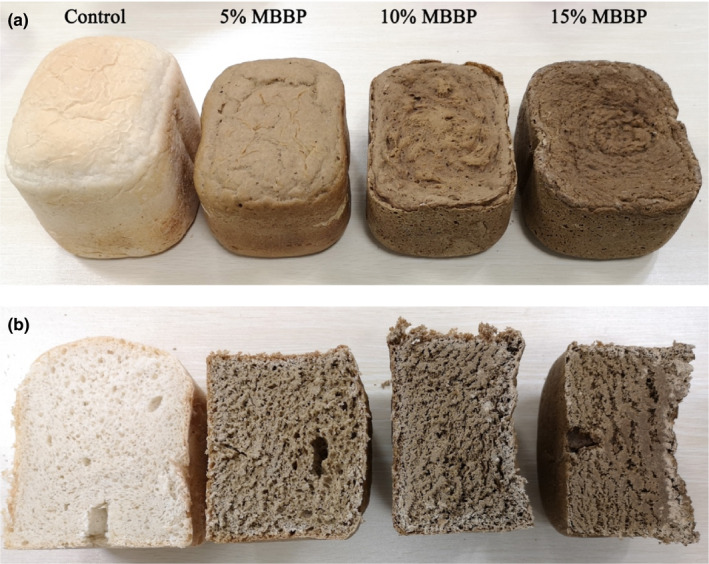
The appearance (a) and section (b) of normal bread and MBBP‐breads

The normal bread, 5%, 10%, and 15% MBBP‐bread were cut into small pieces (prepared as Table 1), then dried at 100°C in the oven. The bread and standard laboratory fodder were put into high‐speed functional grinder to powder, bread powder and standard laboratory fodder powder were mixed with water (bread powder: fodder powder = 1:1) to make a 3–4 cm cylindrical rod. Normal bread fodder, 5% MBBP‐bread fodder, 10% MBBP‐bread fodder, and 15% MBBP‐bread fodder were under test (as in Figure 1, main nutritions were shown in Table 2).

**TABLE 2:** The main nutritions of fodder and four kinds of bread

Fodder	Carbohydrate (%)	Protein (%)	Cellulose (%)	Fat (%)
Fodder	44	18		5
Normal bread	42.6	5.2	–	2.4
5% MBBP‐bread	38.6	4.7	3.3	2.4
10% MBBP‐bread	35.1	4.2	6.6	2.3
15% MBBP‐bread	20.7	3.8	9.9	2.3

### 2.3 Animals experiment

ICR male mice (3–4 weeks) were provided from the Experimental Animal Centre of Soochow University. The mice were raised in standard experimental conditions of humidity of 50%–80%, temperature of 18–25°C, and 12‐hr light/dark cycle. Mice acclimation for 3 days, the mice could free gain normal fodder and purified water. After 3 days of acclimation, all mice (*n* = 60) were randomly divided into three groups: control group (*n* = 10), normal group (*n* = 10), and type 2 diabetes model group (*n* = 40). The control group and the normal group mice were fed with normal fodder. Mice in type 2 diabetes model group received high‐fat and high‐sugar diet for 4 weeks, high‐fat and high‐sugar diet was composed with 26% carbohydrate, 26% protein, and 35% fat. The model group mice were fasted for 12 hr, followed by intraperitoneal injection of low dose of STZ (100 mg/kg BW, STZ was provided by Sigma‐Aldrich Fine Chemicals). Seven days after the injection, mice in model group were fasted for 10 hr and fasting blood glucose (FBG) was determined according to Yin's method (Yin, 2017), mice with more than 7.8 mmol/L of FBG were considered to be diabetic and included in the study. The T2D mice were randomly divided into four groups: diabetes model group, 5%, 10%, and 15% MBBP‐bread diet group. Finally, all the mice were divided into control group, normal group, T2D model group, 5%, 10%, and 15% MBBP‐bread fodder group. The mice in control group were fed with SLF for 7 weeks, the mice in normal group and the model group were fed with normal bread fodder for 7 weeks. The other three groups were fed with different dose MBBP‐bread fodder for 7 weeks (Table 3). The blood glucose and weight of mice were measured from the first to seventh weeks. Blood, pancreas, liver, and kidney were collected for the follow‐up experiments. Whole animal research processes were in accordance with guidelines by the Animal Ethics Committee at Soochow University.

**TABLE 3:** Animal experiments grouping and processing method

Groups	Fodder	STZ injection
Control group	Normal fodder	No
Normal group	Normal bread + normal fodder (1:1)	No
T2D Model group	Normal bread + normal fodder (1:1)	Yes
5% MBBP‐bread	5% MBBP‐bread + normal fodder (1:1)	Yes
10% MBBP‐bread	10% MBBP‐bread + normal fodder (1:1)	Yes
15% MBBP‐bread	15% MBBP‐bread + normal fodder (1:1)	Yes

### 2.4 Oral glucose tolerance test

The glucose tolerance of mice was measured at sixth week. All mice were fasted for 12 hr; FBG was determined by tail‐vein sampling using a glucose meter (0 min). Mice received an intraperitoneal injection of glucose (1 g/kg BW). FBG was determined at appropriate time intervals (30, 60, 90, and 120 min).

### 2.5 Serum Insulin and Glycosylated Hemoglobin (HbA1c) determination

The serum insulin of mice was analyzed by assay kit (Nanjing Jiancheng Bioengineering Institute). HbA1c was analyzed by assay kit (Lai Er Bio‐Tech).

### 2.6 Blood lipid determination

The ADVIA 1,800 Chemistry System (Siemens Ltd.) was used for appraisal of the total cholesterol (CHOL), low‐density lipoprotein cholesterol (LDL‐C), high‐density lipoprotein cholesterol (HDL‐C), and triglyceride (TG) in serum.

### 2.7 Assays of the enzyme activities

The glutathione peroxidase (GSH‐PX) and superoxide dismutase (SOD) in kidney of mice were analyzed by assay kit. The GSH‐PX assay kit (A005), SOD assay kit (A001‐1), and MDA assay kit (A003‐1) were purchased from the Nanjing Jiancheng Bioengineering Institute, Nanjing, China.

### 2.8 Assays of CREA, UREA, ALT & AST

The creatinine (CREA) and serum urea (UREA) in kidney of mice were analyzed by assay kit. CREA assay kit was purchased from Jin‐Pin Chemical Technology (Shanghai) Co., Ltd. UREA assay kit was purchased from Nanjing Jin Yibai Biological Technology Co., Ltd. We analyzed glutamic‐pyruvic transaminase ALT and AST (glutamic oxalacetic transaminase) of mice by assay kit (Nanjing Jiancheng Bioengineering Institute).

### 2.9 Histopathology

The mice were dissected; pancreas, liver, and kidney were collected. The tissue samples were soaked at solution of formalin (10.0%) for 1 week. Samples were dehydrated by acetone. After being embedded in paraffin, samples were cut into 3 µm thickness slices. Samples were colored by hematoxylin and eosin (H&E), and were observed under an optical microscope.

### 2.10 Immunofluorescence assay

Pancreas were collected from the mice. The tissue samples were soaked at solution of formalin (10.0%) for 1 week. Samples were dehydrated by using acetone. After being embedded in paraffin, samples were cut into 6‐µm thickness slices. The primary antibody was incubated with tissues, then second antibody combined with primary antibody. The samples were observed under a fluorescence microscope (DM6000B, Leica Microsystems Inc).

### 2.11 Western blotting analysis

Western blotting was operated according to methods described by Afrin (Afrin et al., 2016). In the liver samples, all protein concentration was measured by BCA assay kit (Beyotime). To analyze protein levels, the protein extracts which were same quality were separated by using 7.5%–15% SDS polyacrylamide gel electrophoresis, then transferred protein from electrophoresis membranes to nitrocellulose membranes. Nitrocellulose membranes were blocked by 5% Bovine Serum Albumin (BSA). Antibodies and reagents were supplied from KeyGEN Tech CO., LTD. incubated overnight at 4℃. Then, the membranes were washed with TBST for three times, each for 5 min. The secondary antibodies which were bound to chemiluminescence agents were incubated for 1.5 h. After that, the membranes were washed again with TBST for 4 times, each for 5 min. G:BOX chemiXR5 was used for imaging.

### 2.12 Statistical analysis

The experimental data were expressed as the Mean ± *SD* and were analyzed with Origin 8.5 software. The one‐way ANOVA was used to evaluate the difference between multiple groups, with *p* < .05 considered as significant and *p* < .01 as very significant.

## 3 RESULTS

### 3.1 Body weight

**FIGURE 2:**
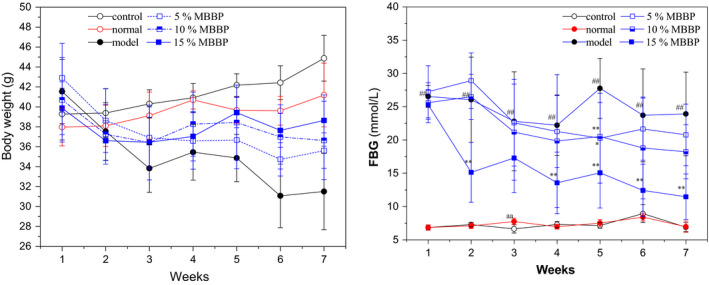
Effect of MBBP‐breads on bodyweight and FBG in T2D mice. aa *p* < .01: versus control group; ^##^ *p* < .01: versus normal group. ^*^ *p* < .05: versus model group. ^**^ *p* < .01: versus model group

Diabetes induced weight loss is a common clinical symptom. Therefore, the weight changes were measured in T2D mice during treatment for 7 weeks. In Figure 2a, mice in the control and the normal group continuously gained weight. The mice in the model group kept losing weight; weight loss was 24.14%. There was a significant change in body weight between the three treatment groups. In consideration of effects on mice weight recovery, 15% MBBP‐bread diet group was best compared with model group. The results showed that MBBP‐bread could effectively restore the weight loss of T2D mice.

### 3.2 FBG

High blood glucose is the most typical characteristic of diabetes; at present, the main diabetes drugs on the market have good blood glucose control ability. Therefore, the FBG of mice was examined for 7 weeks. In Figure 2b, the FBGs of the control group and the normal group remained between 6.85 and 7.75 mmol/L, while model group maintained between 22.21 and 27.76 mmol/L and its blood glucose significantly increased compared with normal group (*p* < .01). When the mice were orally administered MBBP‐bread for 7 weeks, blood glucose in 5%, 10%, and 15% diet group decreased significantly compared to the model group (*p* < .05 or *p* < .01), blood glucose levels fell 23.78%, 28.79%, and 54.66%, respectively. The results showed that MBBP‐bread diets could effectively decreased the blood glucose levels of T2DM mice.

### 3.3 Oral glucose tolerance

**FIGURE 3:**
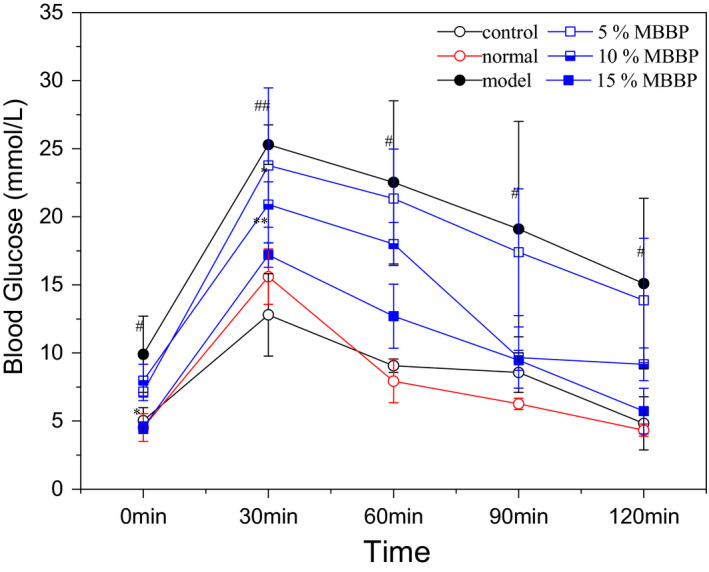
Effect of MBBP‐breads on oral glucose tolerance of T2D mice. ^#^ *p* < .05: versus normal group. ^##^ *p* < .01: versus normal group. ^*^ *p* < .05: versus model group. ^**^ *p* < .01: versus model group

Diabetics have a disordered blood glucose metabolism, and blood glucose regulation is impaired. Oral glucose tolerance test (OGTT) can help researchers to understand the body's ability to regulate glucose level. Therefore, the glucose tolerance was measured in the mice to assess the effect of MBBP bread on blood glucose regulation. In Figure 3, glucose tolerance of control and normal group were ordinary, glucose tolerance of model group severely worse compared to normal group (*p* < .05 or *p* < .01). When the mice were orally administered MBBP‐breads for 7 weeks, the glucose tolerance of mice treated with MBBP‐bread diets recovered to different degrees. The 15% MBBP‐bread diet group was the best glucose tolerance from 17.2 mmol/L at 0.5 hr decreasing to 5.7 mmol/L at 2 hr compared to model group (*p* < .01). The results showed that MBBP‐bread could effectively restore glucose tolerance of T2D mice.

### 3.4 Serum insulin

**FIGURE 4:**
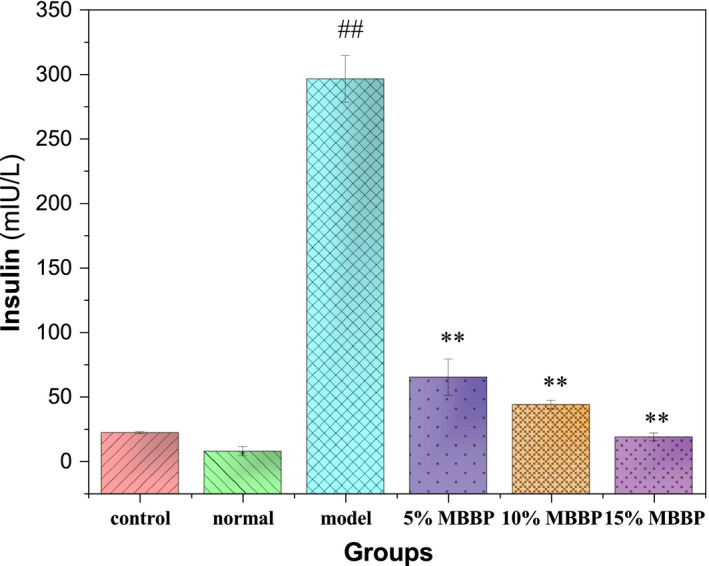
The effect of MBBP‐breads on serum insulin of T2D mice. ^##^ *p* < .01: versus normal group. ^**^ *p* < .01: versus model group

At present, the pathogenesis of type 2 diabetes is mainly divided into two aspects: One is the deficiency of insulin secretion, and the other is the insulin resistance. Hyperinsulinemia and hyperglycemia are also common when insulin resistance occurs (Nakagomi et al., 2013). Therefore, the serum insulin content of the mice was measured. As shown in Figure 4, serum insulin levels were low in the control group and the normal group, ~22 and ~ 8 mUI/L, respectively. The level of the model group was as high as ~ 296 mUI/L, which was significantly higher than that of the normal group (*p* < .01). When the mice were orally administered MBBP‐bread diets for 7 weeks, serum insulin in 5%, 10%, and 15% MBBP‐rich bread diet group decreased significantly compared to the model group (*p* < .01), and there was a significant dose dependence. 15% MBBP‐bread diet group has decreased to ~ 19 mUI/L, which was lower than control level and close to the normal serum insulin level. The results showed that MBBP‐bread could effectively decreased serum insulin content of T2DM mice, and can improve insulin resistance to a certain extent.

### 3.5 HbA1c

**FIGURE 5:**
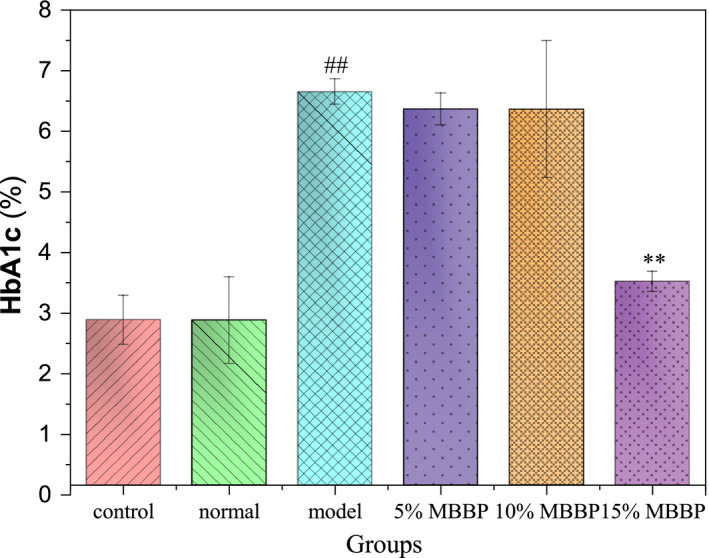
Effects of MBBP‐breads on HbA1c in T2D mice. ^##^ *p* < .01: versus normal group. ^**^ *p* < .01: versus model group

HbA1c is important to a large extent, because it gives clinicians an objective assessment of the effects of glucose control over the past period of time (Goldstein et al., 1986). In addition, the effect of HbA1c has also been proved by clinical diabetes control and complications tests (Kilpatrick et al., 1998). As shown in Figure 5, the HbA1c in the control group was basically the same as that in the normal group, there was no significant difference between the two groups. The content of HbA1c in the model group was double (6.65%) that of the normal group, and there was a significant difference (*p* < .01). When these T2D mice were orally administered MBBP‐bread diets for 7 weeks, the HbA1c of mice treated with MBBP‐bread diet decreased in different degrees, HbA1c of 15% MBBP‐bread diet group decreased to 3.52% significantly compared to model group (*p* < .01), close to the HbA1c level (2.88%) in both normal and control groups. The results showed that high concentration of MBBP‐bread could effectively decreased HbA1c of T2D mice.

### 3.6 Blood lipid

Besides the disorder of blood glucose metabolism in vivo, diabetic patients are also accompanied by the disorder of lipid metabolism in vivo. With the deepening of research, glucose metabolism and lipid metabolism jointly influence the development of diabetes in many aspects. Early in the onset of diabetes, patients with diabetes have a disorder of lipid metabolism. With the deepening of the disease, the abnormality of lipid metabolism is more significant (Turner et al., 1998). According to prior investigations, the main causes of death related to type 2 diabetes are cardiovascular and cerebrovascular complications, and thus, measurement of blood lipids in patients with type 2 diabetes is a critical factor in assessing their risk of cardiovascular and cerebrovascular complications (Li et al., 2012). As shown in Table 4, the TG, CHOL, HDL‐C, and LDL‐C of control group were basically the same as that of normal group, the TG, CHOL, HDL, and LDL conditions of model group were severely worse compared with normal group (*p* < .05 or *p* < .01). When the mice were orally administered MBBP‐bread diet for 7 weeks, the TG, CHOL, HDL, and LDL of mice treated with MBBP‐bread diet decreased in different degree, the TG, CHOL, HDL, and LDL of 15% MBBP‐bread diet group decreased significantly compared to model group (*p* < .05). The results showed that MBBP‐bread has lipid regulation ability to T2D mice in certain degree.

**TABLE 4:** Effect of MBBP‐breads on blood lipids of T2DM mice

Groups	TG(mmol/L)	CHOL(mmol/L)	HDL‐C(mmol/L)	LDL‐C(mmol/L)
Control group	1.49 ± 0.17	2.21 ± 0.28	2.12 ± 0.15	0.21 ± 0.05
Normal group	1.43 ± 0.14	2.45 ± 0.23	2.44 ± 0.12	0.24 ± 0.04
Model group	2.16 ± 0.10^#^	4.43 ± 0.67^#^	3.50 ± 0.86^#^	0.52 ± 0.07^#^
5% MBBP‐bread	1.92 ± 0.40	3.96 ± 0.13	3.03 ± 0.43	0.44 ± 0.02*
10% MBBP‐bread	1.75 ± 0.62	3.24 ± 0.50	2.68 ± 0.43	0.43 ± 0.08
15% MBBP‐bread	1.66 ± 0.20*	3.54 ± 0.42	2.52 ± 0.21	0.43 ± 0.03*

^#^ *p* < .05: versus normal group.

^##^ *p* < .01: versus normal group.

* *p* < .05: versus model group.

### 3.7 Pancreas

#### 3.7.1 Histopathological observation of pancreas

**FIGURE 6:**
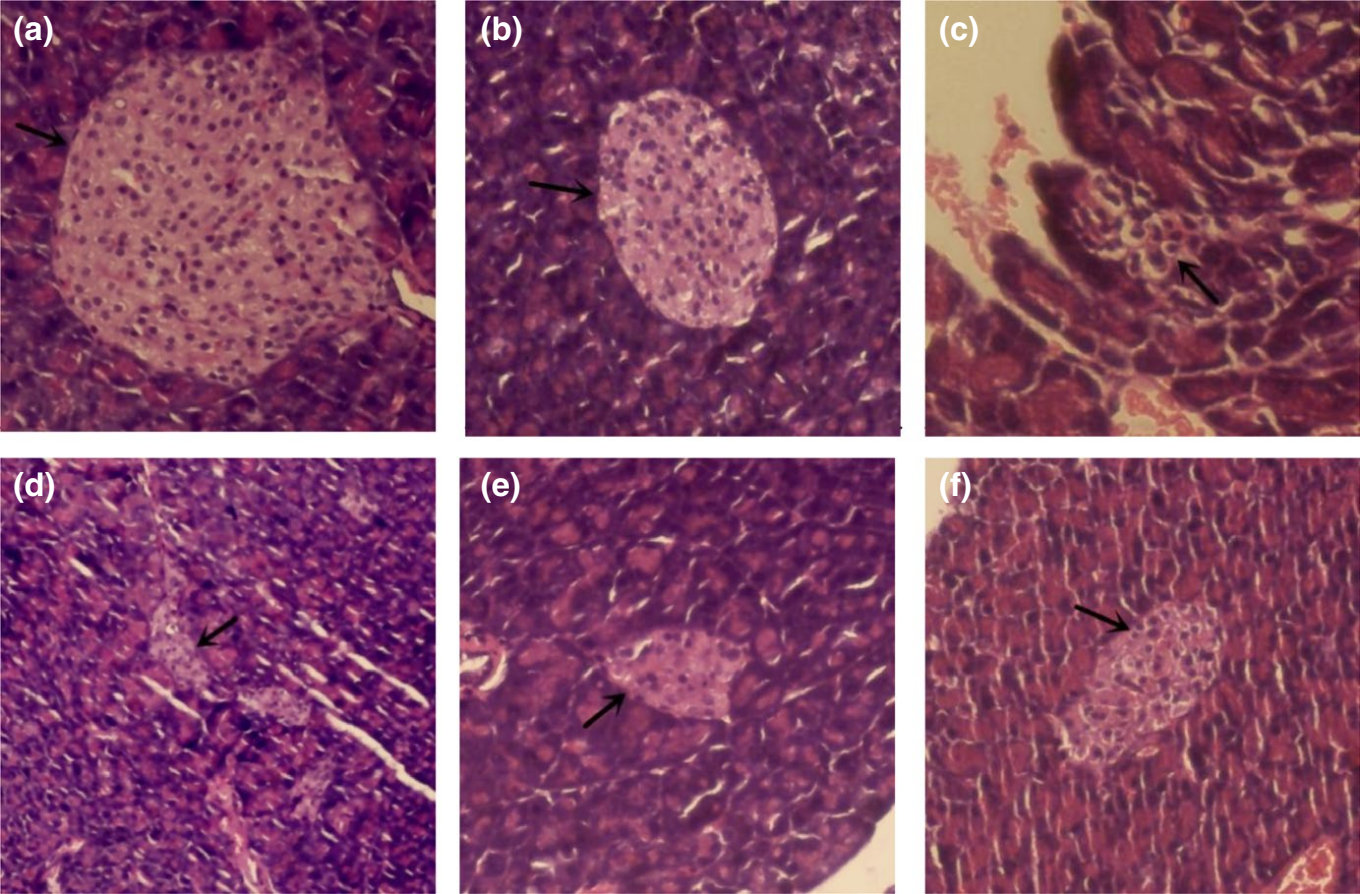
Effect of MBBP‐breads on pancreatic tissue of T2D mice (stain: HE, × 400). (a) Control group; (b) normal group; (c) model group; (d) 5% MBBP‐bread fodder group; (e) 10% MBBP‐bread fodder group; (f) 15% MBBP‐bread fodder group. The arrow shows the islets with beta cells

Damage to islet β‐cells affects the production and secretion of insulin, which in turn causes the body's internal substance and energy metabolism disorders and insulin resistance (Kayaniyil et al., 2010). As shown in Figure 6, pathological changes were not observed in the islets of mice in the control group and normal group. The islet injury of mice in the model group was severe, such as pancreas was small, number of islet cells decreased greatly, cell vacuolation was serious. When the mice were orally administered MBBP‐bread diet for 7 weeks, the pancreas islets of mice treated with MBBP bread gradually recovered, the distribution was even and compact, and the structure was more complete. These results showed that MBBP bread effectively restored the islet injury of T2D mice.

#### 3.7.2 Insulin expression

**FIGURE 7:**
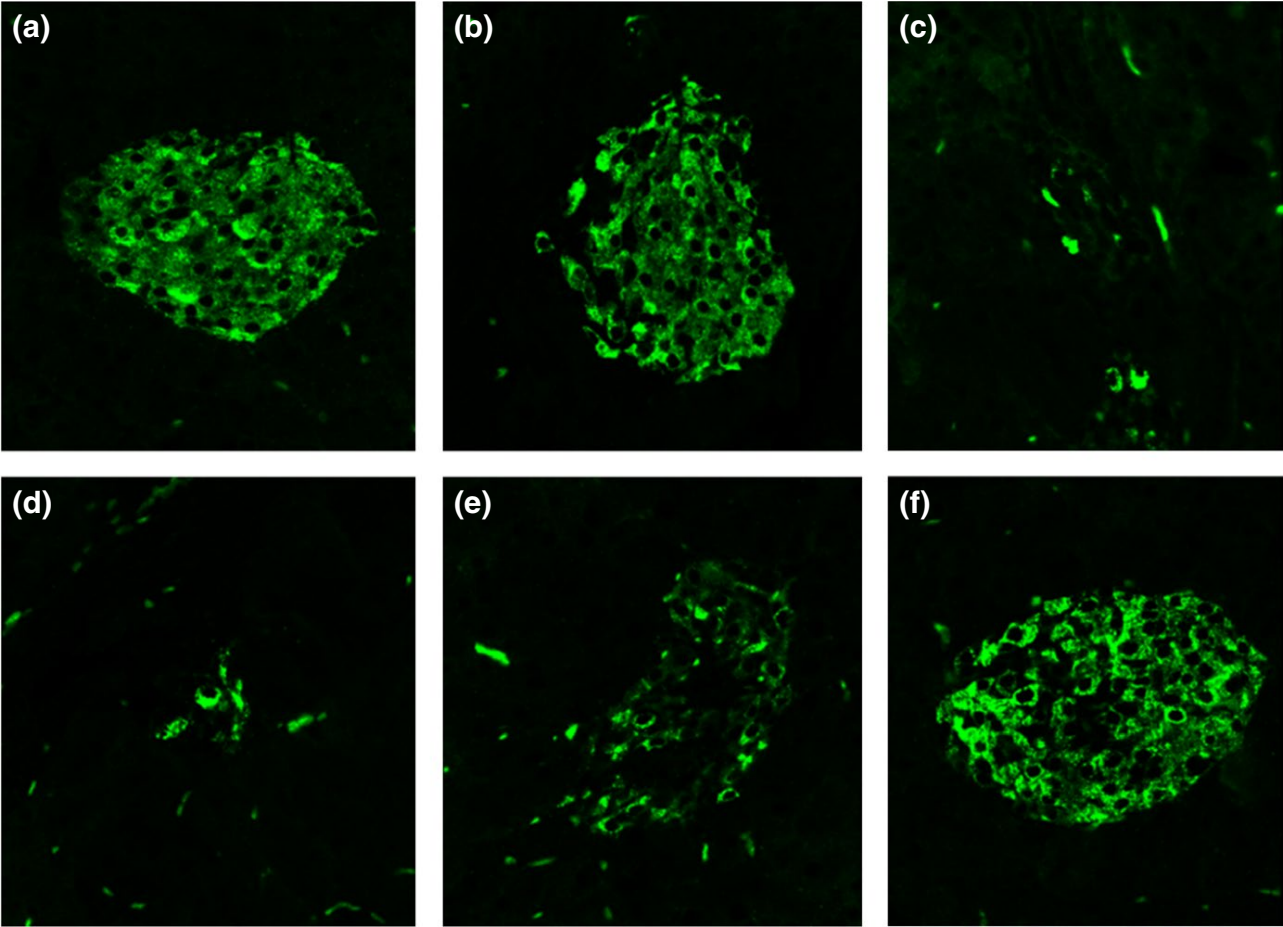
Effects of MBBP‐breads on insulin expression of T2D mice (×200). (a) Control group; (b) normal group; (c) model group; (d) 5% MBBP‐bread diet group; (e) 10% MBBP‐bread diet group; (f) 15% MBBP‐bread diet group

One of the important causes of diabetes is the islet damage in the pancreas, which leads to the disorder of insulin secretion and thus causing diabetes (Norquay et al., 2009). As shown in Figure 7, large amounts of insulin (green fluorescence) were secreted in the islets of the control and normal group. Insulin secretion in the pancreas islets was minimal in model group mice. When the mice were orally administered MBBP‐bread diet for 7 weeks, with the increase of addition, the green fluorescence intensity in the islets gradually became stronger and the structure of the islets became intact, indicating that the number of islets β cells increased and showed a certain dose effect. The results showed that MBBP‐bread effectively restored insulin expression of T2D mice.

#### 3.7.3 PDX‐1 expression

**FIGURE 8:**
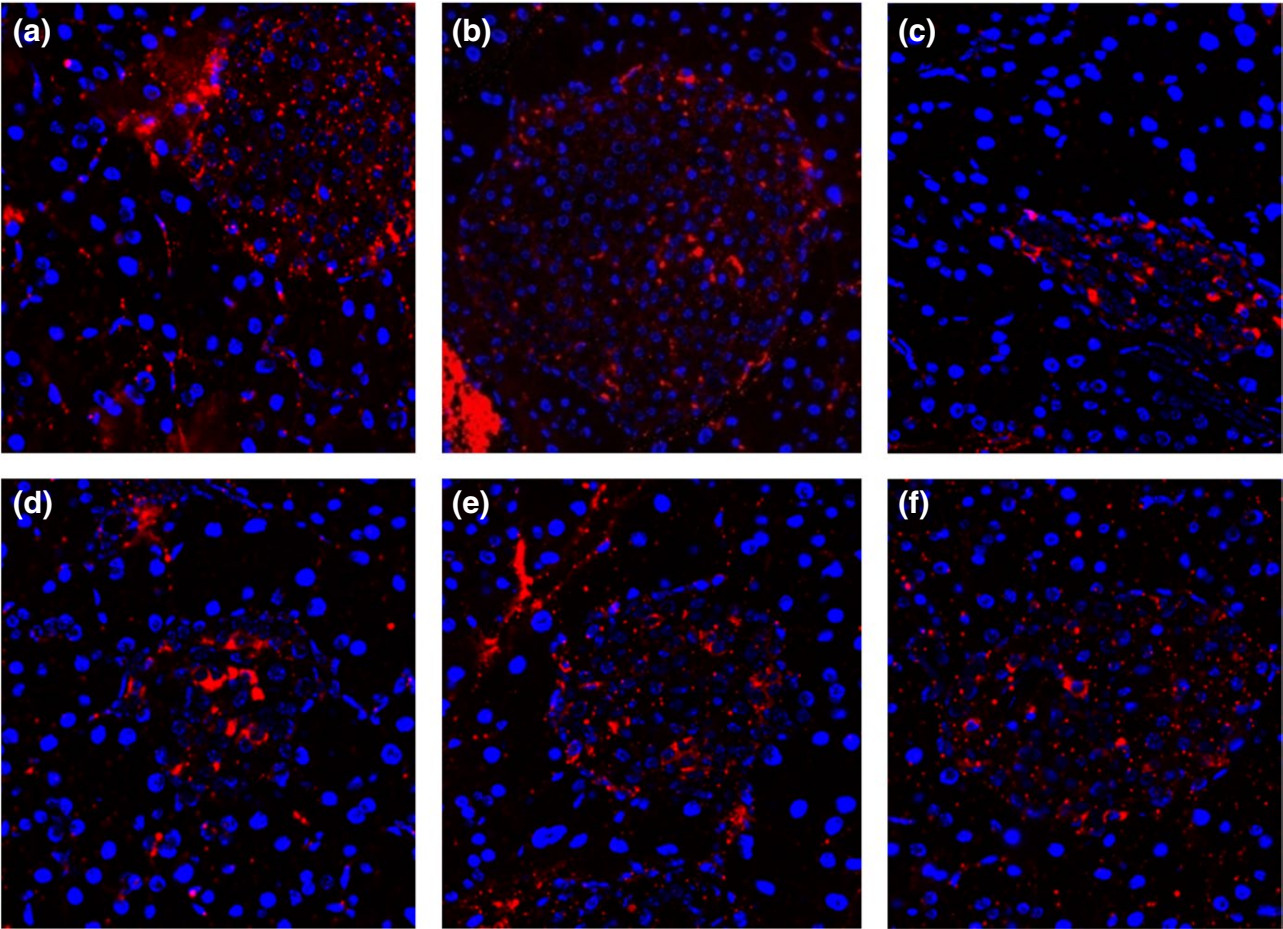
Effect of MBBP‐breads on PDX‐1 expression of T2D mice (×200). (a) Control group; (b) normal group; (c) model group; (d) 5% MBBP‐bread diet group; (e) 10% MBBP‐bread diet group; (f) 15% MBBP‐bread diet group

Pancreatic duodenal homeobox‐1(PDX‐1) plays a key role in the development of pancreatic and islet cells (Mckinnon and Docherty, 2001). Its absence is closely related to the development of type 2 diabetes. As shown in Figure 8, large amounts of PDX‐1 (red fluorescence) were secreted in the islets of the control and normal group. The secretion of PDX‐1 in pancreatic of model group decreased significantly compared to normal group. When the mice were orally administered MBBP‐bread diet for 7 weeks, the amount of PDX‐1 in the islets of mice treated with MBBP‐bread diet increased gradually, and the distribution was consistent with islet structure. The results showed that MBBP‐bread effectively decreased PDX‐1 expression of T2DM mice.

### 3.8 Liver

#### 3.8.1 Liver function

**FIGURE 9:**
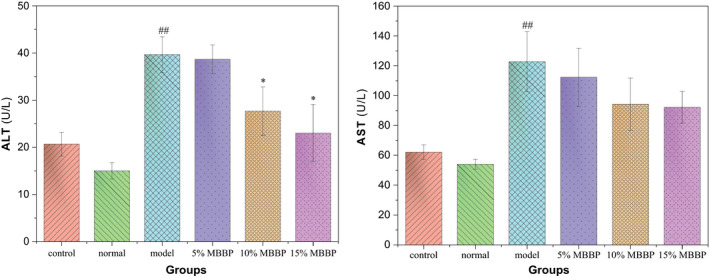
Effect of MBBP‐breads on liver function of T2DM mice. ^##^ *p* < .01: versus normal group; ^*^ *p* < .05: versus model group

Liver disease is one of the important causes of death in type 2 diabetes (Tolman et al., 2007). Liver function is an important index to evaluate liver injury. The determination of liver function is usually done by detecting the content of glutamic‐pyruvic transaminase (ALT) and glutamic oxalacetic transaminase (AST) in the blood. As shown in Figure 9, the ALT and AST in serum of control group were basically the same as that of normal group. The ALT and AST of model group increased significantly compared to normal group (*p* < .01). When the mice were orally administered MBBP‐bread diet for 7 weeks, the ALT and AST of mice treated with MBBP‐bread diet decreased in certain degree. In particular, the ALT of 10% and 15% MBBP‐bread diet groups declined significantly compared to model group (*p* < .05). The results showed that MBBP‐bread effectively restored liver function of T2DM mice.

#### 3.8.2 Liver histopathological observation

**FIGURE 10:**
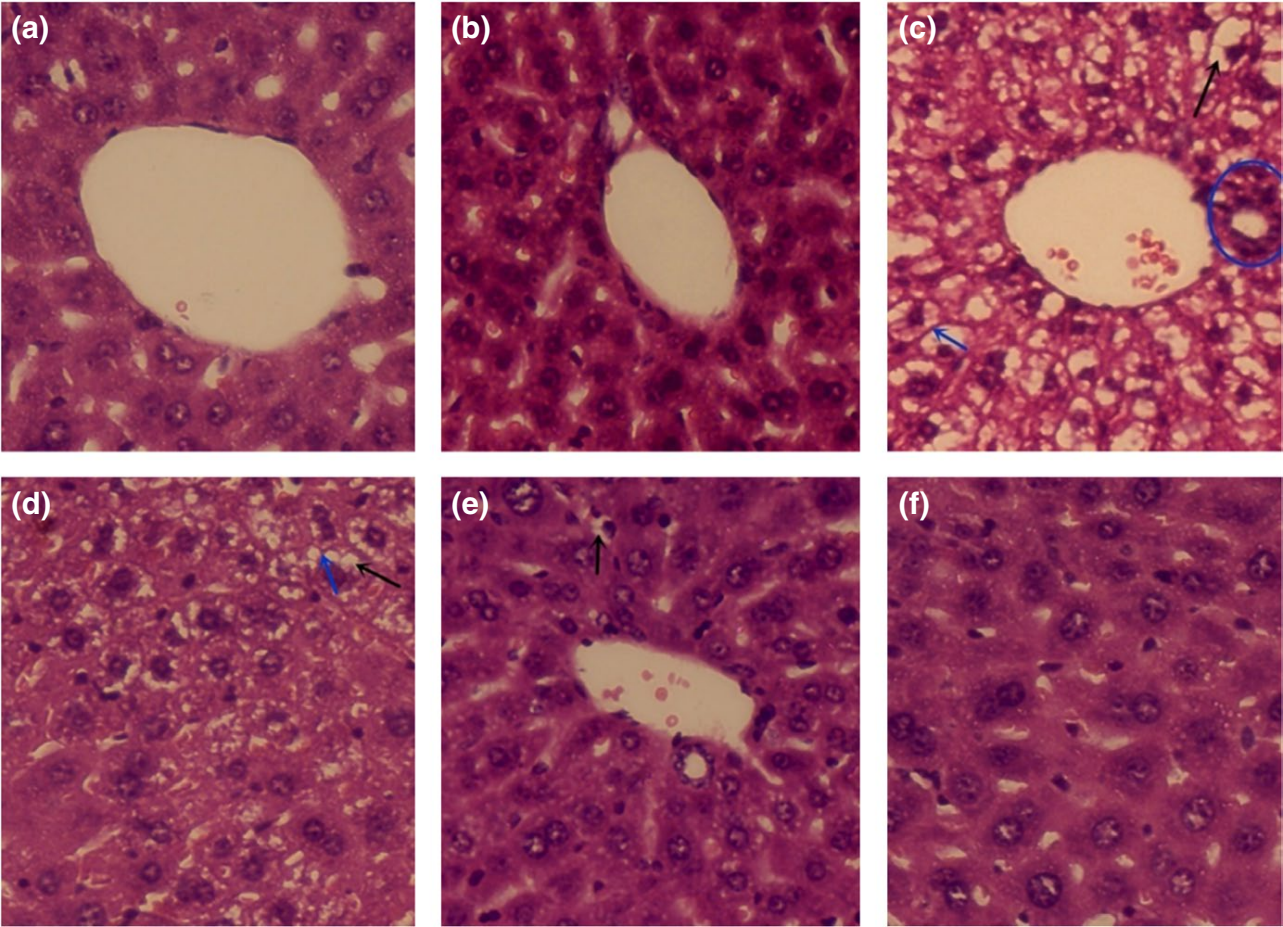
Effect of MBBP‐breads on liver tissue of T2D mice (stain: HE, ×400). (a) Control group; (b) normal group; (c) model group; (d) 5% MBBP‐bread diet group; (e) 10% MBBP‐bread diet group; (f) 15% MBBP‐bread diet group. Blue circle: inflammatory infiltration of cells; Black arrow: cell fat vacuoles; Blue arrowhead: fibrosis

To further evaluate the effect of MBBP‐bread on the liver of T2D mice, the histopathological sections of liver were observed. As shown in Figure 10, pathological changes were not observed in the liver of mice in the control group and normal group. The liver injury of mice in the model group was severe including inflammatory infiltration of cells, cell fat vacuoles, and fibrosis. When the mice were orally administered MBBP‐bread diet for 7 weeks, with the increase of metering, the liver injury of mice treated with MBBP‐bread diet recovered gradually. The results showed that MBBP‐bread effectively restored liver injury of T2D mice.

#### 3.8.3 Signaling pathway

Liver is an important organ of metabolism of glucose for glucose metabolism of liver cells by controlling of insulin and insulin signaling pathways in the cell; the PI3K/AKT insulin signaling pathway is critical for glucose absorbtion and transportation (Li et al., 2015). When the insulin signaling pathway in the liver is damaged, the body's expression of some key proteins changes (Klover and Mooney, 2004). Changes in the insulin signaling pathway of PI3K/ATK are closely related to decrease of insulin sensitivity and the development of diabetes (Kobayashi et al., 2012). Adenosine monophosphate‐activated protein kinase (AMPK) is an heterogenous trimer protein composed of α, β, and γ subunits, AMPK promotes glucose absorption in surrounding tissues by inducing the transfer of glucose transporter isoform 4 (GLUT4) to the plasma membrane and activating GLUT4 gene expression through phosphorylation transcription factors (Mcgee and Hargreaves, 2010), GLUT4's function is to transfer glucose to cells in insulin‐sensitive tissues for utilization, so that cells can normally perform various physiological functions (Mohammad et al., 2002).

**FIGURE 11:**
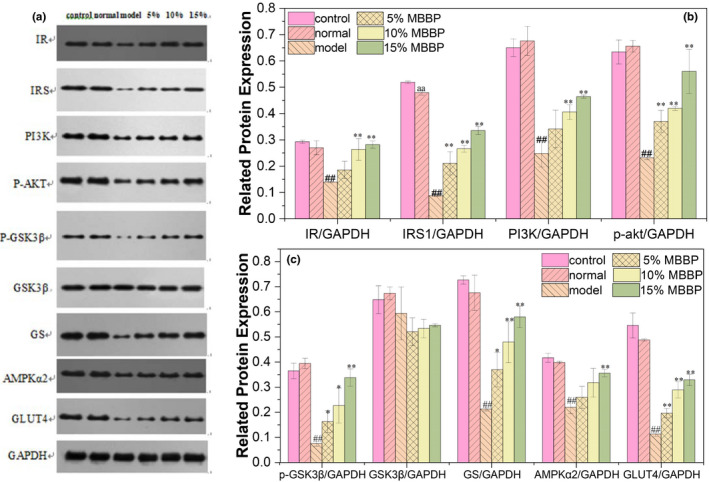
Effect of MBBP‐breads on the key protein expression of PI3K/AKT insulin signaling pathway in T2DM mice liver. (a) Western blot analysis of IR, IRS, PI3K, p‐AKT, p‐GSK3β, GSK3β, GS, AMPKα2, and GLUT4 protein expressions; (b) Quantitative analysis of IR, IRS, PI3K, and p‐AKT protein expressions; (c) Quantitative analysis of p‐GSK3β, GSK3β, GS, AMPKα2, and GLUT4 protein expressions. aa *p* < .01: versus control group; ^##^ *p* < .01: versus normal group; ^*^ *p* < .05: versus model group;^**^ *p* < .01: versus model group

The expression levels of key proteins AMPK_α2_ and GLUT4 in the PI3K/AKT signaling pathway were determined. As shown in Figure 11, there was little difference in protein expression between the control group and the normal group. The protein expression levels of IR, IRS, PI3K, p‐AKT, p‐ GSK3β, GS, AMPK_α2,_ and GLUT4 in the model group significantly decreased compared to normal group (*p* < .01). When the mice were orally administered MBBP‐bread diet for 7 weeks, with the increase of metering, the protein expression level above of mice treated with MBBP‐bread diet increased obviously compared to model group (*p* < .05 or *p* < .01), and showed a certain dose effect. The results showed that MBBP‐bread could effectively activate the insulin signaling pathway and reduce blood glucose by improving the glycogen synthesis in liver of T2D mice.

#### 3.8.4 Hepatic glycometabolism

**FIGURE 12:**
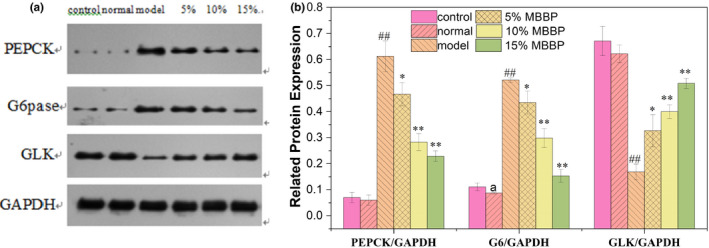
Effect of MBBP‐breads on key protein enzyme expression of glucose metabolism in T2D mice liver. (a) Western blot analysis of PEPCK, G6pase, and GLK. (b) Quantitative analysis of PEPCK, G6pase, and GLK. a *p* < .05: versus control group; ^##^ *p* < .01: versus normal group; ^*^ *p* < .05: versus model group; ^**^ *p* < .01: versus model group

Liver plays its important role in glucose metabolism, the regulation of glucose of liver cells is through gluconeogenesis and glycolysis two ways to achieve dynamic equilibrium of glucose, the two ways play an important role in maintaining the stability of the blood glucose concentration. The expression of gluconeogenesis key enzyme phosphoenol pyruvate carboxyl kinase (PEPCK), glucose‐6‐phosphatase (G6pase), and glycolysis key enzyme glucose kinase (GLK) was determined. As shown in Figure 12, there was little difference in protein expression between the control group and the normal group. The protein expression levels of PEPCK and G6pase in the model group significantly decreased compared to normal group (*p* < .01); the protein expression levels of GLK in the model group significantly increased compared to normal group (*p* < .01). When the mice were orally administered MBBP‐bread diet for 7 weeks, with the increase of MBBP dose, the PEPCK and G6pase expression level of mice treated with MBBP‐bread diet increased obviously compared to model group (*p* < .05 or *p* < .01), the GLK expression level of mice treated with MBBP‐bread diet decreased obviously compared to model group (*p* < .05 or *p* < .01), close to the expression in the normal group. The results showed that MBBP‐bread could effectively decreased the gluconeogenesis effect, increased the glycolysis effect, so that T2D mice blood glucose decreased.

#### 3.8.5 Lipid metabolism and inflammatory factors

**FIGURE 13:**
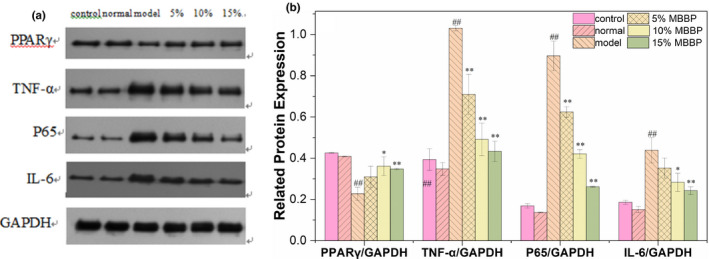
Effects of MBBP‐breads on PPARγ, TNF‐α, P65 & IL‐6 protein expression in liver tissue of T2DM mice. (a) Western blot analysis of PPARγ, TNF‐α, P65 and IL‐6; (b) Quantitative analysis of PPARγ, TNF‐α, P65 and IL‐6; ^##^ *p* < .01: versus normal group; ^*^ *p* < .05: versus model group; ^**^ *p* < .01: versus model group

Peroxisome proliferators activated receptor (PPAR) is primary in the regulation of lipid metabolism; PPARγ is one of PPARs subunits. Inflammation also causes insulin resistance. The expression of PPAR and inflammatory factors P65, TNF‐α, and IL‐6 was measured. As shown in Figure 13, there was little difference in protein expression between the control group and the normal group, the protein expression levels of PPARγ in the model group significantly decreased compared to normal group (*p* < .01), the protein expression levels of P65, TNF‐α, and IL‐6 in the model group significantly increased compared to normal group (*p *< .01). When the mice were orally administered MBBP‐bread diet for 7 weeks, the PPARγ expression level of mice treated with MBBP‐bread fodder increased obviously compared to model group (*p *< .05 or *p *< .01), the P65, TNF‐α, and IL‐6 expression level of mice treated with MBBP‐bread fodder decreased obviously compared to model group (*p *< .05 or *p *< .01) with a certain dose effect. The results indicated that MBBP‐bread diet could effectively improve the lipid metabolism and reduce the inflammation in the liver of T2DM mice.

### 3.9 Kidney

#### 3.9.1 Histopathological observation

**FIGURE 14:**
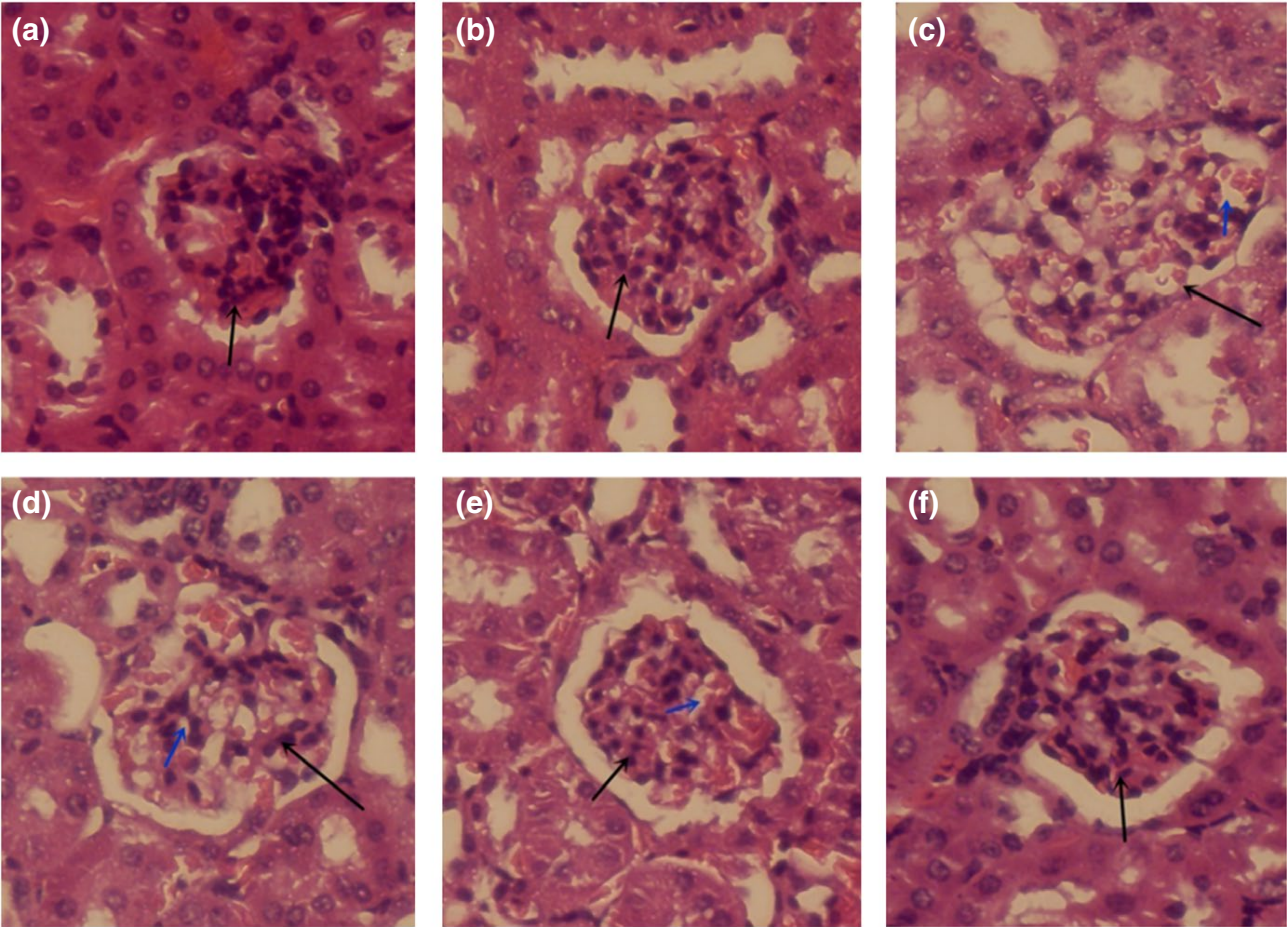
Effect of MBBP‐breads on kidney of T2D mice (stain: HE, ×400). (a) Control group; (b) normal group; (c) model group; (d) 5% MBBP‐bread fodder group; (e) 10% MBBP‐bread fodder group; (f) 15% MBBP‐bread fodder group. Black arrow: glomeruli; Blue arrow: cell vacuolization

To further evaluate the effect of MBBP‐bread on the kidney of T2DM mice, the histopathological sections of mice kidney were observed. As shown in Figure 14, pathological changes were not observed in the kidney of mice in the control group and normal group. The kidney injury of mice in the model group was severe including glomerular volume increases, cell vacuolization, and cell population decline. When the mice were orally administered MBBP‐bread diet for 7 weeks, with the increase of MBBP dose, the kidney injury of mice treated with MBBP‐bread diet improved gradually, 15% MBBP‐bread diet group's kidneys almost recovered. The results showed that MBBP‐bread effectively restored kidney injury of T2D mice.

#### 3.9.2 Kidney function

Diabetic nephropathy is a complication of diabetes, kidney function is an important index to evaluate kidney injury, and the determination of kidney function is usually done by detecting the content of CREA and UREA. As shown in Table 5, there was little difference in CREA and UREA between the control group and the normal group, the CREA and UREA of model group increased compared to normal group (*p *< .05). When the mice were orally administered MBBP‐bread diet for 7 weeks, the CREA and UREA of mice treated with MBBP‐bread diet decreased in certain degree, close to the expression in the normal group. The results showed that MBBP‐bread could decrease CREA and UREA in certain degree.

**TABLE 5:** Effect of MBBP‐breads on CREA and UREA of T2DM mice

Groups	CREA (μmol/L)	UREA (mmol/L)
Control group	14.20 ± 0.44	8.75 ± 0.31
Normal group	13.57 ± 0.42	7.16 ± 0.20
Model group	16.53 ± 2.91	9.27 ± 0.99^#^
5% MBBP‐bread	14.20 ± 0.87	8.46 ± 0.62
10% MBBP‐bread	16.17 ± 1.19	8.04 ± 0.059
15% MBBP‐bread	13.23 ± 0.46	8.01 ± 0.61

^#^ *p* < .05: versus normal group.

#### 3.9.3 Kidney antioxidant

Researchers have shown that antioxidant drugs can significantly delay the progress of diabetic nephropathy by protecting the activity of antioxidant enzymes and inhibiting lipid peroxidation in the kidneys of T2DM mice (Ha et al., 1999). The kidney T‐SOD, GSH‐Px, and MDA of mice were measured. As shown in Table 6, there was little difference in T‐SOD, GSH‐Px, or MDA between the control group and the normal group. The T‐SOD and GSH‐Px of the model group decreased, and the GSH‐Px in particular decreased obviously compared to the normal group (*p *< .05). The MDA of the model group increased obviously compared to the normal group (*p *< .01). When the mice were orally administered MBBP bread for 7 weeks, the T‐SOD and GSH‐Px of mice treated with MBBP bread increased, especially the GSH‐Px in the 15% MBBP bread diet group that increased obviously compared to the model group (*p *< .05). The MDA of the mice treated with MBBP bread decreased, especially the GSH‐Px of the 15% MBBP bread group that decreased obviously compared to the model group (*p *< .05). The results indicated that MBBP bread could effectively enhance the antioxidant capacity of the kidney in T2D mice.

**TABLE 6:** Effect of MBBP‐breads on antioxidant abilities of kidney in T2D mice

Groups	T‐SOD (U/mgprot)	GSH‐Px (U/mgprot)	MDA (nmol/mgprot)
Control group	132.43 ± 6.39	1,254.60 ± 116.43	1.99 ± 0.30
Normal group	143.41 ± 14.83	1,164.80 ± 40.18	2.21 ± 0.04
Model group	122.78 ± 27.95	997.04 ± 69.71^#^	3.59 ± 0.45^#^
5% MBBP‐bread	139.12 ± 8.98	1,003.81 ± 29.88	2.99 ± 0.07
10% MBBP‐bread	148.08 ± 1.65	1,106.58 ± 57.37	2.77 ± 0.56
15% MBBP‐bread	149.25 ± 19.71	1,116.57 ± 17.58*	2.39 ± 0.18*

^#^ *p* < .05: versus normal group.

* *p* < .05: versus model group.

## 4 DISCUSSION

In this paper, the therapeutic effect of MBBP‐bread on T2D mice was investigated. It is well known that diabetes is a chronic metabolic disorder. The most typical clinical symptoms are weight loss and elevated blood sugar in vivo. Therefore, the therapeutic effects of MBBP‐bread on T2D mice could be reflected by blood glucose level and weight recovery. The results showed that the MBBP‐bread effectively reduced the blood sugar of the T2D mice up to 54.66%, and the weight loss of the MBBP‐bread group was only 30% of the model group. It was quite clear that MBBP‐bread could effectively slow down the weight loss of T2D mice, and balanced the blood glucose level of T2D mice.

Striking features of type 2 diabetes are insulin resistance and insulin secretion reduce. Insulin resistance means that the body is not sensitive to insulin, the generation of normal insulin dose cannot maintain normal biological effects, namely the insulin‐sensitive cells mediates glucose uptake and disposal to insulin resistance. Some scholars believed that insulin resistance has appeared in early onset of type 2 diabetes, to maintain normal blood glucose levels, the islet beta cells continuously increase the secretion of insulin to compensate, and the insulin secretion after reaching the maximum level will decrease. The vicarious insulin secretion not compete with insulin resistance results in diabetes. It can be seen that insulin resistance is a key point during the occurrence and development of type 2 diabetes, so relieving insulin resistance of diabetes is a key step in the treatment of diabetes.

Our results showed that the abnormal increase of serum insulin level of T2D mice was significantly decreased after MBBP‐bread treatment for 7 weeks, indicating that MBBP‐bread could effectively relieve hyperinsulinemia. In addition, we studied on serum insulin levels in T2DM mice. After taking in a large amount of normal bread contained plenty of carbohydrates, T2D mice serum insulin levels were very high. On the one hand, it indicated that excessive consumption of carbohydrate diet was harmful to T2D mice. On the other hand, the experimental results also proved that MBBP‐bread could eliminate the adverse effects on diabetic patients, so that diabetic patients were able to take it as diets safely. Beside the determination of serum insulin, we calculated HOMA‐IR and ISI, and further concluded that feeding on MBBP‐bread for a long period could significantly increase insulin sensitivity and improve insulin resistance to help to alleviate T2D.

Oral glucose tolerance test is very important to understand the islet's regulation of blood glucose in vivo. According to the experimental results, after MBBP‐bread treatment for 7 weeks, T2D mice showed significant improvement in glucose tolerance, indicating that MBBP‐bread effectively restored the islet regulation on blood glucose in T2D mice. The detection of HbA1c is a key indicator in evaluating the therapeutic effect on diabetes. HbA1c is synthesized from sugars and hemoglobin in a nonenzymatic reaction, and accumulates throughout the life cycle of red blood cells. The rate of synthesis is related to the blood sugar concentration in the environment of red blood cells. The detection of HbA1c could reflect the long‐term mean blood glucose level. When the percentage is high, the diabetes condition is serious. The results showed that the HbA1c level of T2D mice was effectively reduced after 7 weeks’ feeding on MBBP‐bread, which proved that MBBP‐bread had significant therapeutic effect on T2D mice.

Dyslipidemia is a common symptom in diabetes mellitus, which can cause lots of diabetes complications, including coronary heart disease, hypertension, and atherosclerosis (Mitra et al., 1995). Diabetes drugs should have the effect of blood lipid regulation. The results showed that MBBP‐bread could improve the dyslipidemia of T2DM mice.

The pancreas is the most important organ in the pathogenesis of diabetes. The treatment of the diabetic pancreas is mainly reflected in two aspects: restoring the islet function and preventing the apoptosis of the islet cells. HE staining is used to observe the pancreas of mice in each group. The islet size of T2D mice increased after the MBBP‐bread treatment. The results showed that the MBBP‐bread effectively repaired the islet injury of T2D mice. The islet is the organ that secretes insulin, and it is mentioned earlier that insulin secretion decreases in diabetes. In the experiment, the insulin secretion of each group was observed by immunofluorescence, and the results showed that the MBBP‐bread could effectively restore the insulin secretion function of the pancreas of T2D mice. PDX‐1 is a significant transcription factor in the development of pancreas orientation. In addition, PDX‐1 can continuously inhibit apoptosis of islet cells. The results of immunofluorescence experiments showed that the expression of PDX‐1 in the pancreas could be improved effectively.

Liver is also a main place for insulin resistance since its role in glucose and lipid metabolism. Glucose metabolism in the liver is regulated to insulin and its insulin signaling pathway. Insulin resistance in the liver is characterized by impaired insulin signaling pathways in the liver cells (Cordero‐Herrera et al., 2015). So it is of great value to evaluate the liver injury of diabetes mellitus. First liver function index is associated with liver injury, alanine transaminase (ALT) and aspartate aminotransferase (AST) are major indicators of liver function. The results showed that the MBBP‐bread could significantly decrease ALT and AST level in the T2D mice, which proved that MBBP‐bread effectively restored the T2D mice liver function. The HE staining results showed that the liver cells of T2D mice had complete structure and homogeneous cytoplasm after MBBP‐bread treatment. So that MBBP‐bread could effectively repair liver damage in T2D mice. Besides, we investigated the internal mechanism of the effect of MBBP‐bread on liver of T2D mice. PI3K/AKT is a golden insulin signaling pathway, and the substrate is GSK3β, which is the key to glycogen synthesis. p‐AKT inactivated its phosphorylation and GS activity increased, which in turn increased glycogen synthesis and decreased blood glucose. In the model group, the expression levels of IR, IRS, PI3K, p‐AKT, p‐GSK3β, and GS decreased. After the treatment with MBBP‐bread, the expression levels of these proteins were significantly increased. By activated PI3K/AKT insulin signaling pathway, GLUT4 was also significantly up‐regulated. This suggested that the MBBP‐bread could transfer glucose to the liver by activating GLUT4 and improve the blood glucose regulation of T2D mice.

The regulation of blood glucose in liver is mainly through two metabolic pathways of glycosylation and glycolysis. We studied the expression of two key enzymes PCK and G6pase in glycosylation, and GLK in glycosylation. The results showed the expression of PCK and G6pase was increased while GLK was decreased in the diabetic model group. After MBBP‐bread treatment, the situation reversed. The results showed that the MBBP‐bread could effectively reduce the liver's gluconeogenesis and increase the glycolysis to decrease the blood glucose.

In diabetic patients, lipid metabolism is disordered, PPARγ also plays an important role in the regulation of lipid metabolism, with decreased expression in insulin resistance. In study of T2D mice, both the expression of PPARγ was down‐regulated in liver, and both the expression of PPARγ was up‐regulated after the treatment of MBBP‐bread. The results showed that the MBBP‐bread could effectively improve the lipid metabolism in the liver of T2DM mice. Meanwhile, the inflammatory factors in the liver of each group were also analyzed. Results showed that the MBBP‐bread effectively alleviated the inflammatory response in the liver of T2D mice.

Diabetic nephropathy is a common complication of diabetes. We measured CREA and UREA in serum, which are both important for diagnosing early renal disease in clinic. The results showed that MBBP‐bread could effectively reduce the content of CREA and UREA in the serum and improve the renal function of T2D mice. The rise of oxidative stress is common in diabetic patients, and oxidative stress injury is important to promote diabetic nephropathy. We measured the contents of T‐SOD, GSH‐Px, and MDA in the kidney of mice in each group. The contents of T‐SOD and GSH‐Px decreased and MDA increased in the diabetic model group. After treated with MBBP‐bread, the contents of T‐SOD and GSH‐Px increased and MDA decreased in the kidney of T2D mice. The results indicated that MBBP‐bread could improve the oxidative damage of kidney of T2D mice to some extent. Further HE staining was used to observe the kidney injury in T2D mice. The results showed that the kidney injury of T2D mice was significantly improved after treated with MBBP‐bread, indicating that MBBP‐bread can effectively improve the kidney injury of T2D mice.

## CONFLICT OF INTEREST

The authors would like to declare that they have no conflict of interest.

## ETHICAL APPROVAL

Ethics approval was not required for this research.

## Data Availability

The data that support the findings of this study are available from the corresponding author upon reasonable request.
